# Gestational diabetes mellitus and obstetric outcomes in a Ghanaian community

**DOI:** 10.11604/pamj.2019.32.94.17334

**Published:** 2019-02-27

**Authors:** Ahmed Tijani Bawah, Robert Amadu Ngala, Huseini Alidu, Mohammed Mustapha Seini, Joshua Dokurugu Kwame Wumbee, Francis Agyemang Yeboah

**Affiliations:** 1 Department of Medical Laboratory Science, School of Allied Health Sciences, University of Health and Allied Health Sciences, Ho, Ghana; 2 Department of Molecular Medicine, School of Medical Sciences, Kwame Nkrumah University of Science and Technology, Kumasi, Ghana; 3 Laboratory Department, Greater Regional Hospital, Accra, Ghana; 4 Quality Control Unit, Quality Control Directorate, Kumasi Technical, University, Kumasi, Ghana

**Keywords:** Gestational diabetes mellitus, stillbirth, cesarean operation

## Abstract

**Introduction:**

This study was aimed at evaluating effect of Gestational diabetes mellitus (GDM) and maternal characteristics on pregnancy outcome. GDM has several risk factors including; advanced maternal age, ethnic background, obesity and family history of diabetes mellitus. These pregnancy complications are associated with fetal morbidity and mortality and may lead to macrosomia and shoulder dystocia. Others are stillbirth, miscarriages, preterm and small for gestational age babies.

**Methods:**

This was a retrospective case-case control study which compared maternal characteristics and pregnancy outcome among pregnant women with and without GDM. Diagnosis of GDM was done in accordance with the American Diabetes Association (ADA) criteria. Weight and height were determined and Body mass index (BMI) calculated. Pregnancy outcome was determined at the end of pregnancy and information on maternal characteristics obtained using questionnaire and patient folders.

**Results:**

Those who developed GDM were significantly older (OR= 1.772; 95% CI =1.432-2.192; P<0.0001) and had higher BMI (OR=1.637; 95% CI=1.004-1.289; P=0.044) than those who did not. A significant number of those who developed GDM also had stillbirths OR= 5.188; 95% CI=1.093-24.613; p=0.038) and cesarean deliveries (OR=14.362; 95% CI=3.661-56.335; p= 0.001).

**Conclusion:**

Women who develop GDM are more likely to deliver stillborn or macrosmic babies and may require surgical intervention in order to have normal deliveries.

## Introduction

This paper looks at obstetric outcomes in relation to GDM. GDM is the occurrence of hyperglycemia in pregnant women who have never had diabetes. This condition is becoming more common as a result of the increasing prevalence of type 2 diabetes mellitus (T2DM) and obesity [1]. Women who have had GDM are likely to have it again in later pregnancies. Women with GDM may develop T2DM later on; however, reducing weight, eating balanced diet and getting adequate exercise will minimize their risk of getting diabetes mellitus (DM). Screening GDM women for DM after birth, can detect women at risk of developing DM [2]. The link between neonatal morbidity and diabetes during pregnancy had been recognized since the early 1940s [3, 4] and by the 1950s GDM had been documented [5]. In the 1960s it was reported that the degree of hyperglycemia during pregnancy could be associated with diabetes after pregnancy and subsequently a criteria was established for diagnosing GDM [6]. The prevalence of GDM is increasing globally [7] and varies from country to country. In Australia, it ranges from 5.2% to 8.8% [8] and in the United States it is 1% to 14%, [9]. GDM is responsible for almost 90% of all pregnancies complicated by glucose intolerance [9]. In Nigeria, the incidence of diabetes mellitus among pregnant women was 1.7% with pre-gestational diabetes accounting for 39% while GDM was responsible for 61% of cases [10] and in Ghana a prevalence of 10% was reported at the Korle-Bu Teaching Hospital [11].

Risk factors for GDM are; a previous diagnosis of GDM, family history indicating a first degree relative with type 2 diabetes mellitus, poor previous obstetric outcome, previous macrosomia and advanced maternal age [12]. Others are; obesity, polycystic ovarian syndrome, glycosuria during pregnancy and ethnic background [13]. Smoking during pregnancy was also demonstrated to double the risk of GDM [14]. One of the effects of GDM is; the risk of developing type 2 DM in the future (Kim *et al.* 2002). About 10% of GDM patients develop DM soon after delivery [15] and about 70% develop diabetes mellitus between 5 to 15 years [16]. The risk is higher in women who required insulin for treatment [17]. Women with GDM are likely to develop type 2 diabetes mellitus in later life [16], and children of women with GDM have a higher risk for childhood and adult obesity and an increased risk of type 2 diabetes later in life [18]. Women with GDM are also at risk of developing preeclampsia and have a higher risk of cesarean deliveries [16]. There is also the risk of abruptio placentae, preterm labour and postpartum uterine atony [19]. Furthermore, women with GDM were reported to experience twice the number of urinary tract infections than the controls. This is due to the increased amount of glucose in the urine during pregnancy which can cause pyelonephritis and asymptomatic bacteriuria [19]. It has therefore been recommended to check glucose tolerance in all women who have had GDM at six weeks post-partum and every three years afterwards [20]. Over the past decades, several researchers have reported on various effects of GDM on infant's health and these include; an increased risk of macrosomia, birth injuries such as shoulder dystocia [21]. The incidence of GDM is increasing worldwide which may be due to changing life style and delay in childbirth as a result of delay marriages which may be as a result of the fact that more girls are attaining higher educational status, hence this study sought to examine maternal characteristics, obstetric and family history of participants and to elucidate the effect of these parameters on pregnancy outcomes.

## Methods

This was a retrospective case-control study involving 200 participants; 80 women with GDM and 120 women without GDM at the Volta Regional Hospital, Ho, Ghana from January 2014 to December 2016. Information on maternal characteristics such as age, BMI, family history of diabetes mellitus, number of previous miscarriages and stillbirth were obtained from patient folders. Participants whose antenatal records indicated that they had preexisting diabetes were excluded. Also excluded were those with hemoglobin A1c level ≥6.5% at first antenatal visit. All pregnant women in this facility are screened for GDM between 24 and 28 weeks of gestation where the women's plasma glucose is tested after a 50g oral glucose load. When the plasma glucose measured after one hour is ≥7.8 mmol/l, the woman is referred to do oral glucose tolerance test (OGTT). Diagnosis of GDM is usually made after 24 weeks of gestation using the results of the OGTT based on the criteria of the American Diabetes Association [22].

Postnatal records were also reviewed and information on pregnancy outcome was recorded for each participant and the number of stillbirths as well as live births documented. For those who had live births, they were subdivided into those who had normal spontaneous vaginal deliveries and assisted deliveries comprising; cesarean section, misopristol (cytotec) insertion and a combination of cytotec and episiotomy. The study protocol was reviewed and approved by the joint Committee on Human Research Publication and Ethics of the School of Medical Science, Kwame Nkrumah University of Science and Technology and the Komfo Anokye Teaching Hospital, Kumasi, Ghana.

**Statistical analysis:** Data were first entered into Microsoft Office Excel 2007 and GraphPad Prism 3.02 program was used to analyze the data. univariate and multivariate logistic regression were used to compare obstetric outcomes among those with GDM and those without GDM. The level of statistical significance was set at P<0.05 for all tests and at 95% confidence interval.

**Ethics approval and consent to participate:** Ethical clearance (Ref CHRPE/AP350/14) was obtained from the joint Committee on Human Research Publication and Ethics of the School of Medical Science, Kwame Nkrumah University of Science and Technology and the Komfo Anokye Teaching Hospital, Kumasi. Participation in this study was voluntary and informed consent was obtained from each study participant.

## Results

In this study, advanced maternal age (OR=1.772; 95% CI=1.432-2.192; p<0.0001) and high BMI (OR=1.637; 95% CI=1.004-1.289; p=0.044) showed strong relationship with GDM (There was also a strong influence of previous cesarean operation (OR=14.362; 95% CI=3.661-56.335; p<0.0001) and family history of diabetes (OR=2.692; 95% CI=0.953-7.608; p=0.043) on the development of GDM. Parity, previous miscarriages, previous stillbirth and number of pregnancies did not show significant difference between those who developed GDM and those who did not (Table 1). When the pregnancy outcomes (Stillbirth, live birth, spontaneous abortion and induced labour) were stratified by GDM (Table 2), there were no significant differences in deliveries through cesarean operation (p=1.000) and deliveries with the help of misopristol (Cytotec) (p=0.507) between the GDMs and the non-GDMs.

**Table 1:** Multivariate logistic regression of maternal characteristics and family history of participants stratified by GDM

Variable	Regression coefficient (β)	OR (95% CI)	P value
Age	0.572	1.772 (1.432-2.192)	0.000
BMI (Kg/m^2^)	0.129	1.637 (1.004-1.289)	0.044
RWD	0.99	2.692 (0.953-7.608)	0.043
NC	0.077	1.08 (0.977-1.194)	0.192
MC	1.169	3.218 (0.882-11.745)	0.029
SB	1.176	3.243 (0.828- 12.701)	0.091
CS	2.665	14.362 (3.661-56.335)	0.000
NP	-0.348	0.706 (0.2-2.495)	0.589

RWD=Relative with diabetes mellitus, NC=Number of children (Parity), MC= Number of previous miscarriages,

SB=Number of previous stillbirths, CS=Number of previous caesarean operations,

NP=Number of pregnancies, BMI= Body mass index.

**Table 2:** Multivariate logistic regression of factors associated with pregnancy outcomes among the study respondents with GDM

Variable	Regression coefficient (β)	OR (95% CI)	P value
SB	1.646	5.188 (1.093-24.613)	0.038
NSVD	0.131	1.14 (1.015-1.28)	0.027
CS	2.665	14.362 (3.661-56.335)	0.000
C	-0.403	0.668 (0.2-2.23)	0.512
C+E	2.8	16.449 (4.5-60.128)	0.005

SB= Stillbirth, NSVD= Normal spontaneous vaginal delivery, CS= Caesarean section, C= Cytotec, C+E= Cytotec and Episiotomy

However, there were significant differences in the deliveries which were conducted with the use of a combination of Cytotec and episiotomy (OR=16.449; 95% CI=4.5-60.128; p=0.005) as well as delivery of stillborn babies (OR=5.188; 95% CI=1.093-24.613; p=0.038) between those with GDM and those without GDM. Analysis of post-partum maternal characteristics on birth weight (Table 3) showed that there was no significant difference in the birth weight resulting from sex of baby, and the method by which delivery was achieved. There was, however, strong influence of baby's weight by GDM (OR=14.2; 95% CI=4.35-46.00; p<0.0001) indicating that women who developed GDM are 14.2 times more likely to deliver macrosomic babies than those did not develop GDM.

**Table 3:** Univariate analyses of post-partum maternal characteristics on birth weight

Variables	OR (95% CI)	p-value
Sex of Baby	1.4 (0.47 - 4.10)	0.553
Assisted Delivery	Ref	
Caesarean section	0.01 (0.002 - 0.13)	0.0001
Cytotec	1.0 (0.10 - 9.61)	1
Cytotec + Episiotomy	0.1 (0.01 - 1.92)	0.13
Still birth	1.0 (0.03 - 29.81)	1
GDM	14.2 (4.35 - 46.00)	0.0001

OR-odds ratio; 95%CI-95% confidence interval; Ref-reference variable; GDM-gestational diabetes mellitus

## Discussion

The mean first trimester BMI of those who developed GDM was significantly higher than those without GDM. This is in consonant with a study which indicated that the risk of developing GDM amplified about 3 fold in obese women [12] but contrary to a study in which there was no significant difference in the BMI of women with GDM and the controls and that pregnancy weight gain was a better indicator of pregnancy outcome [23]. This study also revealed that poor previous obstetric outcome was related to the development of GDM in later pregnancies due to the fact that significant number of the GDM group had previous cesarean operations, suggesting that they might possibly have delivered macrosomic babies in those previous pregnancies. This is in agreement with a study done in Madrid [24], which found macrosomia to be more common in children of mothers who had developed gestational diabetes mellitus. Another study also reported that macrosomia was more common in children of women who had developed diabetes mellitus after gestational diabetes mellitus [25].

In GDM, the fetus is persistently affected by high glucose levels from maternal circulation. This leads to elevated level of fetal of insulin (maternal insulin itself cannot cross the placenta). The anabolic effects of insulin can lead to macrosomia. [26]. The mean age of the GDM group was significantly higher than their peers. The relationship between advanced maternal age and GDM was clearly demonstrated by this study and it is in agreement with an earlier study [13]. In a study done in urban Iranian population, women with GDM had a higher rate of stillbirths than those with normal glucose tolerance [27] which is similar to this present study. Similarly our study reports higher number of cesarean operation among those with GDM as compared to those with normal glucose tolerance which is in consonant with a previous study which reported a higher number of cesarean deliveries among women with GDM as a result of the fact that their infants are normally large for gestational age and often have higher risk for prematurity [28]. The prematurity associated with pregnancies complicated by glucose intolerance may be responsible for higher number of stillbirths among the GDMs.

## Conclusion

Women who develop GDM are more likely to deliver stillborn or macrosmic babies and may require minor or major surgical intervention through episiotomy and cesarean operation respectively. Obese women and women with previous cesarean operation are prone to the development of gestational diabetes mellitus during pregnancy. The findings in this study reemphasize the need to intensify screening of pregnant women for GDM and to institute measures to reduce the effects of this condition on the baby and the mother.

### What is known about this topic

- Women who are obese are likely to develop GDM during pregnancy;
- Women who have relatives with diabetes are at risk of developing GDM.

### What this study adds

- Women with GDM are more likely to deliver with the help of cytotec and episiotomy;
- More women with GDM deliver stillborn babies compared to women without GDM.

## Competing interests

The authors declare no competing interests.
